# Decoding Complex Traits in Goats Through Genome-Wide Association Studies: Progress, Challenges, and Perspectives

**DOI:** 10.3390/ijms27072945

**Published:** 2026-03-24

**Authors:** Da Feng, Chen Wei, Si-Yi Hu, Shang-Quan Gan

**Affiliations:** College of Coastal Agricultural Sciences, Guangdong Ocean University, Zhanjiang 524088, China; fda_1020@163.com (D.F.); weichenwjf@126.com (C.W.);

**Keywords:** genome-wide association study, goat, economic traits, candidate genes, genetic variation

## Abstract

Goats play a significant role in the global livestock industry, with breeders aiming to investigate genetic variations linked to crucial economic traits for enhancing production performance. Genome-wide association studies (GWASs) are a highly effective method for identifying the associations between complex traits or diseases and genetic variations in goat at the whole-genome level. By analyzing large datasets of goat genomes, GWASs can offer valuable insights into the identification of genetic variations related to key economic traits in goats and aid in the discovery of new genetic variants. These discoveries hold the promise of improving the efficiency of goat production by molecular breeding strategies. This study reviews the fundamental theories and developmental processes of GWAS, focusing on its role in identifying potential genetic loci or genes associated with major economic traits in goats. Additionally, it delves into the challenges involved in unraveling the genetic architecture of complex traits in goats through GWAS and investigates future opportunities for progress to advance the goat molecular breeding.

## 1. Introduction

Goat is an ancient and versatile creature that serves as a primary source of both meat and milk, with its domestication tracing back to the Fertile Crescent region over 10,500 years ago [1]. Currently, it has disseminated across the globe adapting to and becoming endemic in diverse regions [2]. The extensive diversity among goat breeds, while posing challenges for intensive breeding efforts, also underscores the considerable potential of goat genetic resources for future exploration and utilization. However, the goat industry’s drive to enhance production performance to meet global demand faces several challenges, such as overreliance on a single breed, inadequacies in the breeding system, and sluggish progress in goat breeding progress [3].

The myriad challenges confronting the goat breeding industry have sparked a heightened emphasis on the application of advanced breeding techniques specifically tailored for goats. Conventional methods based on phenotypic selection are increasingly inadequate for modern, large-scale breeding programs due to long generation intervals and slow genetic gains [4]. With advances in molecular technology and new biological analyses, researchers have established ways to incorporate genetic variation associated with traits into breeding at the DNA level. Nevertheless, a major hurdle remains the rapid and efficient identification of correlations between genetic variants and phenotypic traits. To some extent, precise localization of quantitative trait loci (QTLs) can help address this issue. Historically, linkage mapping—based on the analysis of parental and offspring populations—has been widely used for QTL identification [5]. However, the inherent constraint of measurability confined to select groups, coupled with the prerequisite for substantial group sizes, poses significant limitations on the advancement of link-age mapping methodologies [6]. Genome-wide association studies (GWAS) [7] are highly favored due to their precision in targeting specific genetic variants and the extensive breadth of their action across the entire genome.

GWASs have played a critical role in uncovering genetic variants influencing complex traits in goats. To data, GWASs have successfully pinpointed 51 distinct complex traits and 971 corresponding genetic signals [8], providing valuable insights into the genes and biological pathways underlying these traits in goats. They have also illuminated the complex interplay of pleiotropy and polygenic effects underlying trait variation in goats [9]. The rapid progress of GWAS in goats has been driven by continuous advances in genomic technologies. Already in 2010, the International Goat Genome Consortium (IGGC; www.goatgenome.org) (accessed on 2 December 2025) inaugurated the innovative Goat SNP50 BeadChip (Illumina Inc., San Diego, CA, USA) [10]. Subsequently, the new generation sequencing (NGS) technology triumphantly accomplished the de novo sequencing of goats, marking a significant milestone in the field of goat genomics [11]. In contrast to microarrays, which are limited to the detection of single nucleotide polymorphisms (SNPs), whole genome sequencing (WGS) offers a broader spectrum of capabilities, encompassing the identification of copy number variations and insertions, thereby offering a more comprehensive understanding of the genetic landscape [12]. Despite these technological advances, GWASs have inherent limitations. They statistically identify associations between genetic variants and phenotypes, but some variants appear significant only due to linkage disequilibrium (LD) with causal mutations and do not directly contribute to trait formation. Moreover, the majority of GWAS signals reside in non-coding regions, complicating the interpretation of their functional impact.

Empirical evidence has demonstrated the utility of GWAS in identifying trait-associated variants, and their application in goat research has grown substantially [13]. However, many studies are constrained by insufficient sample sizes and complex population stratification, often halting at the identification of statistical associations without functional validation or biological interpretation. This has, to some extent, led to inconsistencies across studies. Furthermore, genomic resources for goats remain less comprehensive than those available for model organisms and major livestock species, limiting the mapping resolution and accuracy of GWAS. Given the heterogeneity of existing GWAS in goats and the limitations of current genomic resources, a comprehensive synthesis of the literature is urgently needed. To date, however, no systematic review specifically focused on goat GWAS has been published. Therefore, this review aims to construct a systematic analytical framework to synthesize existing findings, clarify discrepancies arising from methodological differences, and evaluate how insufficient genomic resources constrain mapping accuracy and biological interpretation.

Through a meticulous search of PubMed, we identified articles on GWAS in goats, highlighting the emerging application in the field of GWAS in goat genomics. In this review, we present a comprehensive overview of the screening methodologies employed for genetic variation, fine mapping and the in-depth analysis of associated genes in goats. Additionally, we discuss the anticipated challenges and promising avenues for the utilization of GWAS in the field of goat genetics.

## 2. GWAS: A Successful Tool for Analyzing Goat Genomics

**Figure 1:**
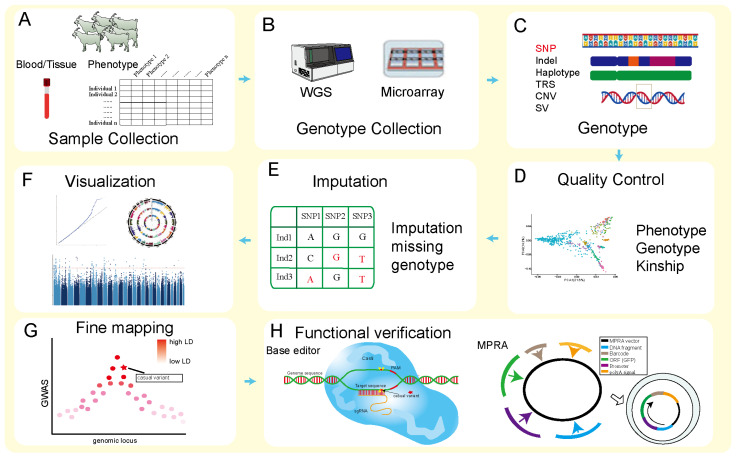
The comprehensive experimental methodology outlining the general procedure for conducting GWAS in goats. (**A**) Blood sample collection for goat samples. (**B**) Sequencing and genotyping through WGS and microarray. (**C**) Processing genotype data to obtain various types of genetic variations. TRS: Tandom repeats sequencing. Indel: Insertion-deletion. CNV: Copy number variation. SV: structure variation. SNP: single nucleotide polymorphism. (**D**) The obtained genotype data requires quality control. (**E**) Correlation analysis between genotype data after quality control and collected phenotype data using statistical models. (**F**) Visualization of GWAS results using software such as R (v4.4.2). (**G**) We need to fine mapping the correlated signals and explore the causal variation mechanism through Mendelian randomization combined with co localization analysis. LD: Linkage Disequilibrium. (**H**) We can perform functional validation through base editing and MPRA methods. PAM: Protospacer Adja-cent Motif. sgRNA: single guide RNA. MPRA: massively parallel reporter as-say. ORF: Open Reading Frame.

GWASs are a robust statistical approach that identifies significant associations between genetic variants and traits across the entire genome. The standard GWAS pipeline involves several key steps (Figure 1). The collection of samples and phenotypic data in goats is challenging, primarily in terms of high labor costs and low standardization of phenotypic recording (Figure 1A). Genotyping is typically performed via either WGS or microarrays (Figure 1B). The wide variety of goat breeds poses additional challenges, as SNP arrays optimized for commercial breeds may introduce ascertainment bias when applied to other populations. Following alignment of sequencing data to the goat reference genome, various forms of genomic variants can be identified; however, research on non-SNP variants in goats remains limited (Figure 1C). The resulting genotypic and phenotypic data undergo rigorous quality control. Given the low accuracy of phenotypic records in goats, quality control parameters require particularly careful adjustment (Figure 1D). Due to the limited variant coverage of genotyping arrays, genotype imputation is performed to infer missing genotypes (Figure 1E). Imputation accuracy in goats is highly dependent on the availability of appropriate reference panels, which are less well-established than those for cattle or pigs, posing a substantial challenge for improving genomic coverage. GWAS is then conducted using a mixed linear model (MLM) to account for population stratification, with results visualized in Manhattan and quantile-quantile (Q-Q) plots (Figure 1F). However, because many GWAS signals in goats are derived from populations with strong family structures or limited LD decay, the scope of these signals is often extensive, necessitating more extensive fine-mapping (Figure 1G). Fine-mapping aims to prioritize putative causal variants from association signals. This step is particularly critical in goats, as the extent of LD can vary considerably across breeds, further complicating the identification of truly causal variants. Finally, the lead variants identified by GWAS largely represent statistical associations and still require functional validation. Although base editing tools and massively parallel reporter assays demonstrate considerable potential for functional validation, their application in goat research remains limited (Figure 1H).

### 2.1. Sample Size and Sample Selection

A well-powered GWAS begins with determining an adequate sample size, as larger cohorts improve the precision and reliability of the findings. Many studies have demonstrated that an exceedingly large sample size has helped mitigate the adverse effects arising from inaccurate phenotype data, particularly the inclusion of unsuitable phenotype features or human errors during the measurement phase [14]. Moreover, for traits with low heritability (e.g., many reproductive traits), substantially larger samples are required to detect statistically significant associations [15]. For GWAS in goats, we selected animals that were as unrelated as possible, based on pedigree records or farmer knowledge [16,17]. This strategy reduces spurious associations caused by population structure and LD from recent shared ancestry [18], and it limits the masking of true trait associations by background genetic similarities among closely related individuals.

Additionally, acquiring extensive phenotypic data for goats poses significant challenges. On one hand, the multitude of goat breeds coupled with limited population sizes for each breed complicates data collection. Simultaneously, the majority of GWASs in goats have concentrated on individual breeds, with sample sizes often below 1000 animals [19,20,21]. On the other hand, collecting data on goat traits like growth, reproduction, and carcass traits requires many specialized technicians or researchers, leading to high costs and yielding limited data. In this context, machine vision technology has demonstrated remarkable capabilities, underscoring its potential to revolutionize various industries and ap-plications [22]. Qin et al. utilized the Sheep Body Size Collector (SBC) to collect phenotypic data and estimate genetic parameters related to growth traits in sheep, thereby enhancing insights into the genetic foundations of these economically significant attributes [23]. Importantly, detection of causal/functional variants among GWAS is directly correlated with the magnitude of the sample size [24], a phenomenon that has been amply illustrated through the utilization of the UK Biobank [25]. Given the inherent challenges in assembling extensive goat databases, it is prudent to prioritize data acquisition and subsequent result analysis under conditions characterized by a relatively modest sample size.

### 2.2. Phenotype

**Figure 2:**
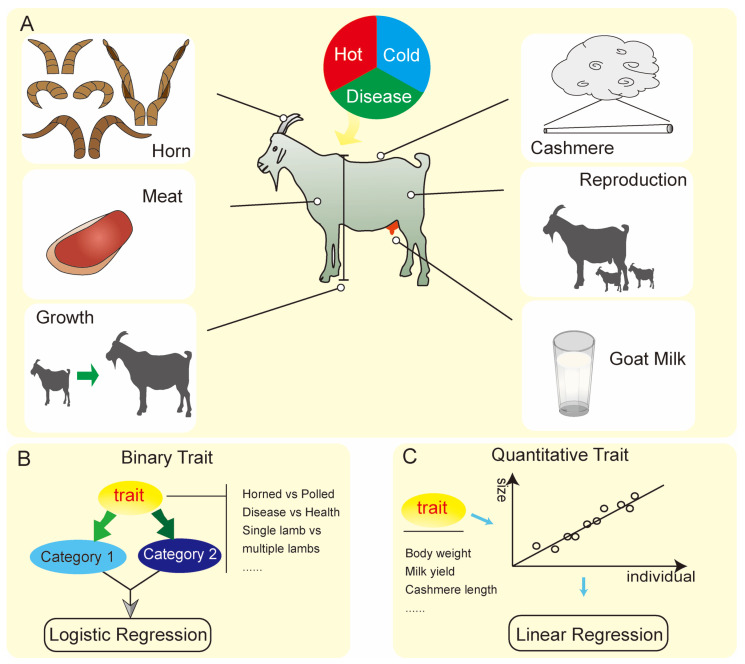
Investigating the diverse phenotypic traits of goats and the corresponding processing methodologies within the framework of GWAS. (**A**) Phenotypic classification of goats. On the left-hand side, are horn traits, meat pro-duction and quality traits, and growth and development traits; On the right-hand side, are cashmere production and quality traits, reproductive traits, milk production and quality traits; The above displays the resistance traits of goats to cold or hot environments and diseases. (**B**) The presence or absence of goat horns, as well as the strength of reproductive ability and other binary traits, are generally analyzed utilizing logistic regression in GWAS. (**C**) Linear regression method is used for GWAS analysis of quantitative traits such as body weight, milk production, and cashmere production.

Given the disparities in goat production capabilities, we conventionally adopted distinct nomenclatures for categorizing goats into meat-producing, dairy-yielding, and cashmere-producing varieties. This categorization encompasses five fundamental traits, including growth and development, milk production, cashmere production, reproduction, and other related traits (such as disease resistance and adaptability) (Figure 2A). According to statistics, GWAS Atlas (https://ngdc.cncb.ac.cn/gwas/) (accessed on 2 December 2025) website has compiled 51 traits of goats currently studied by GWAS [26]. To translate these traits into quantifiable and discernible phenotypes, it is imperative to identify exemplary manifestations of trait expression and subsequently transform them into tangible data points. According to different characteristics, the obtained goat phenotype data can be divided into binary variables and continuous variables. Binary variables refer to phenotypes that classify individuals into two distinct categories. A common example is disease status, where animals are categorized as affected (coded as 1) or healthy (coded as 0). Statistical modeling frequently employs logistic regression as the primary analytical tool (Figure 2B). On the other hand, Continuous variables encompass phenotype data that exhibit continuity and adhere to a normal distribution pattern, with statistical models commonly adopting linear regression as the primary analytical tool (Figure 2C). In the realm of phenotype data processing, two prevalent issues arise. Firstly, outliers (phenotype values that deviate significantly from the normal distribution) are liable to emerge during the processing phase, necessitating their exclusion based on a rigorous assessment of their effect size. Secondly, in the case of samples sourced from diverse locations, the phenotype data may be contaminated by environmental influences, thus necessitating the application of a generalized linear model to disentangle and mitigate these effects.

### 2.3. Genotype

The success of GWAS in uncovering genotype–phenotype associations depends critically on data quality. Advances in sequencing technology have enabled the use of SNP arrays, WGS, and whole-exome sequencing (WES) to generate high-quality genotype data. In goats, the SNP array-based approach is the most widely used and has the most extensive literature, largely due to its cost-effectiveness and speed [27]. A key advantage of WGS and WES is their ability to comprehensively identify variants across the entire genome or exome. Currently, SNP-based GWAS remains the predominant method, although this may change as sequencing costs continue to decline.

The first stage of quality control requires rigorous data verification, highlighting the critical importance of confirming the consistency between the phenotype and genotype data of every individual. On one hand, it is easy to verify if the gender predicted solely from genotype data aligns with the actual gender of the corresponding individual. On the other hand, in scenarios where pedigree information records are available, data validation can be effectively facilitated by utilizing the inferred kinship relationships among individuals [28]. In addition, the handling of genotype data requires a considerably more sophisticated quality control process compared to that needed for phenotype data. The standard quality control protocols for genotype data encompass the following key steps: the elimination of SNPs exhibiting low detection rates (i.e., less than 98–99%), the exclusion of SNPs with infrequent minor allele frequencies (i.e., less than 5%), and the pruning of SNPs that manifest deviations from the Hardy–Weinberg equilibrium (i.e., *p* < 0.0001). These criteria were assessed using PLINK (v1.9.0) software [29].

### 2.4. Addressing Population Structure in Goat GWAS Through the Application of Linear Mixed Models

Population structure has serious implications for goat GWAS, which may lead to a spurious association [30]. The conventional approaches for correcting population structure primarily involved the application of genome control (GC) [31] alongside the utilization of principal component analysis (PCA) [32]. Yu et al. successfully integrated population structure and relative phylogenetic relationships of samples within a mixed model framework, resulting in the derivation of a novel MLM [33]. GWAS conducted in goats frequently incorporated kinship matrices to alleviate the confounding effects of population structure. Importantly, MLM was the most commonly used algorithmic model in goat GWAS, which incorporates kinship matrices to alleviate the confounding effects of population structure [34]. The MLM equation is expressed as:

Y=Wα+Xβ+Zu+e

where Y is the vector of phenotypic observations, α is the effect of the SNP tested for association, W is a vector containing the SNP genotype; β is the vector of the fixed effects, X is the incidence matrices assigning observations to fixed effects; u is the vector of the remaining polygenic effect with $u\sim N(0,G\sigma_{u}^{2})$, where G is the genomic-based relationship matrix (GRM) calculated as $G=\frac{MM}{2\sum p_{i}(1-p_{i})}$; M is the centered genotype matrix, $p_{i}$ is the allele frequency at i-th SNP, and $\sigma_{u}^{2}$ is the additive genetic variance of polygenic effects; Z is the incidence matrix for u; and e is a vector of residual effects with $e\sim N\left(0,I\sigma_{e}^{2}\right)$, where I is an identity matrix and $\sigma_{e}^{2}$ is the residual variance.

The incorporation of kinship matrices in MLM has been widely adopted in goat GWAS to control population stratification arising from the species’ complex breed structure and varying degrees of genetic relatedness. Due to the large number of goat breeds, GWASs often include an excess of individuals with diverse genetic backgrounds, thereby increasing computational demands [35]. This computational burden has driven the development of optimized algorithms for GWAS, as summarized in Table 1. BLINK (v1.0) software and Farm CPU (v1.2.0) software exhibited remarkable performance in mitigating false positives for traits exhibiting medium to high heritability within the goat population [36]. Although numerous GWAS models have been developed and applied in goat research, the choice of model involves inherent trade-offs among computational speed, statistical rigor, and the ability to account for the complex population structure characteristic of goat breeds. Researchers must carefully consider these factors based on the specific population and genetic background under investigation.

### 2.5. Bayesian GWAS

MLM introduces random effects, which can account for a large proportion of the known genetic variance. However, in GWAS, the number of markers generally far exceeds the sample size, which can lead to severe overfitting in traditional multiple linear regression. The Bayesian regression framework provides an effective solution to this issue. It incorporates prior distributions to impose statistical constraints, leading to stabilized estimation of effect sizes for all genetic markers [52]. Bayesian GWAS builds upon the linear mixed model by specifying prior distributions for all unknown parameters. A flat prior is used for the fixed effects β, and conditional on the residual variance, $\sigma_{e}^{2}$, a normal distribution with null mean and covariance matrix ${R\sigma }_{e}^{2}$ is used for the vector of residuals, where R is a diagonal matrix. The prior assumptions for the marker effects α differ across various Bayesian methods. In BayesA, the prior assumption is that marker effects have identical and independent univariate-t distributions each with a null mean: $\alpha_{i}|\sigma_{\alpha_{i}}^{2}\sim N(0,\sigma_{\alpha_{i}}^{2})$. The variance parameter $\sigma_{\alpha_{i}}^{2}$ for each marker is assigned a scaled inverse chi-square prior [53]; BayesB not only permits marker effect sizes but also explicitly assumes that a proportion of markers have zero effect: $\alpha_{i}|\pi ,\sigma_{\alpha_{i}}^{2}\sim \left\{\begin{array}{c} 0\\ N(0,\sigma_{\alpha_{i}}^{2}) \end{array}\right.$. The parameter π denotes the prior probability of a marker having zero effect. The variance parameter $\sigma_{\alpha_{i}}^{2}$ for each marker is assigned a scaled inverse chi-square prior. In a study on milk production traits in dairy goats, a comparison between Bayesian GWAS and traditional GWAS showed that Bayesian GWAS enhances the precision of QTL mapping [54].

### 2.6. Multiple Testing Corrections

In goat GWAS, population stratification is pronounced due to the species’ substantial breed diversity, making multiple testing correction a critical step for identifying significant signals. The traditional Bonferroni correction is known to be highly conservative. The calculation formula is: $\alpha_{adj}=\alpha /m$. Where $\alpha_{adj}$ is the adjusted significance threshold after Bonferroni correction, α is the original significance level, m is the number of independent tests performed. Although this method rigorously controls the family-wise error rate, it can lead to an inflated false negative rate a limitation that becomes more pronounced in goat studies with relatively small sample sizes. Furthermore, determining the appropriate number of independent tests (m) in goats is not straightforward: LD patterns vary considerably across breeds and genomic regions, and the widely used threshold of 5 × 10^−8^, originally derived from human data, may not be directly transferable to goats. An overly stringent threshold risks missing true associations, particularly for polygenic traits. Consequently, many goat GWASs have adopted the false discovery rate (FDR) approach, which serves as a comparatively permissive means of correction and can help mitigate false negatives [55]. However, a more lenient approach may generate an excess of candidate signals, thereby increasing the difficulty of prioritizing causal variants. Currently, goat studies often employ both methods in parallel to capture both large- and small-effect signals; alternatively, the choice of correction method can be tailored to the specific study design and prior knowledge of trait architecture.

## 3. GWAS Provides a New Perspective for Understanding the Quantitative Traits of Goats

Based on the latest information from the International Goat QTL Database (https://www.animalgenome.org/cgi-bin/QTLdb/CH/index) (accessed on 2 December 2025), a total of 1501 QTLs related to various goat traits have been identified [56]. QTL helps us comprehend the genetic structure of complex traits, involving multiple genes, regulatory pathways, and environmental factors. In contrast to typical QTLs that influence a single trait, certain individual QTLs also play a role in regulating multiple traits. For example, Jiang et al. identified a QTL strongly associated with udder depth, fore udder attachment and rear udder attachment in New Zealand dairy goats through a GWAS [57]. In a previous study, Megan also discovered the same QTL affecting fat yield, protein yield, and milk volume in New Zealand dairy goats via a GWAS [54]. This QTL has been consistently identified across multiple studies, supporting its reliability as a functionally important locus. However, the observed differences in associated traits may suggest the presence of either a pleiotropic gene or a cluster of tightly linked genes within this region that simultaneously influence both the anatomical basis of lactation and production performance. This QTL, closely related to milk production traits, is located on chromosome 19 (24–29 Mb) and encompasses 340 variations within a 5 Mb region. The extensive physical interval encompassed by the QTL, which harbors an abundance of genes, necessitates further refinement through fine mapping [58]. The wide physical interval of this QTL reflects a common limitation in current goat GWAS: due to the constraints of SNP chip density and reference genome completeness, the mapping resolution of these studies remains substantially inadequate. This limitation makes it difficult to determine whether the signal within this region originates from a single pleiotropic locus or from the cumulative effects of multiple adjacent loci. Furthermore, neither study explicitly reported the effect size of this QTL or its confidence interval, precluding an assessment of the variant’s actual contribution to the traits and making it impossible to evaluate whether effect estimates were inflated due to small sample sizes. Future studies involving larger, multi-breed populations are warranted to re-estimate the effect size of this locus, thereby assessing its stability across diverse genetic backgrounds and its potential value in breeding applications. This predicament profoundly illustrates the current challenges in goat QTL research: although we have repeatedly mapped important genomic regions, the path from QTL intervals to causal genes and functional mechanisms remains protracted due to insufficient mapping resolution, ambiguous effect sizes, and the lack of cross-population validation.

The expression patterns of QTLs are intricately interconnected with gene expression profiles. Furthermore, the co-localization of these QTLs with GWAS signals can help in unraveling the underlying mechanisms of non-coding variations that contribute to trait alterations [59]. Co-localization, as a determinant of whether a single variation within a locus concurrently influences both GWAS signals and expression quantitative trait loci (eQTLs), provides an effective approach for identifying potentially causal variations mapped onto the genome, generating strong association signals [60]. From a multi-omics perspective, utilizing expression genome-wide association study (eGWAS), which expands the phenotypic scope of traditional GWAS to include gene expression levels, offers a more comprehensive and nuanced examination of the intricate molecular regulatory network underlying genetic variation [61]. The integration of GWAS and eGWAS facilitates an in-depth exploration of the biological pathways underpinning genetic variation and the intricate mechanisms governing trait expression [62]. Recent research has also vali-dated the significance of integrating the aforementioned two approaches for in-depth analysis of the genetic mechanisms underlying complex traits in live-stock and poultry [63,64,65].

### 3.1. Modeling Genetic Effects on GWAS

The genetic variance of quantitative traits can be partitioned into additive, dominant, and epistatic effects. Additive effects refer to the effects of multiple gene loci on a quantitative trait being independent and linearly additive; Dominant effects refer to the interaction between two alleles at the same gene locus, where the phenotype of the heterozygote deviates from the average of the phenotypes of the two homozygous parents [66]; Epistatic effects refer to the irregular genetic effects resulting from interactions between different gene loci [67]. Epistatic effects are not independent but are influenced by other genomic factors, making it extremely difficult to dissect the genetic architecture of traits. In contrast, additive and dominant effects can be predicted and modeled due to their inherent regularity. The standard GWAS model estimates additive genetic effects, thereby capturing the additive component of phenotypic variation. Although a model focusing solely on dominance effects can reveal relevant genetic architectures, most current GWAS implementations do not account for them [68]. However, dominant effects are widely prevalent and play important roles in mammals. For example, Cui et al. [69] found that the dominant effect accounts for one-quarter of the genetic variance in all physiological traits in populations of pigs, rats, and mice. Studies in goats have also demonstrated the important role of dominance effects. For instance, in an investigation of a key SNP influencing milk yield and lactose percentage in Norwegian goats, the dominance effect was found to be significantly larger than the additive effect [70]. Compared to traditional models, the adoption of the additive-dominant effect model in GWAS enables the identification of dominant effect loci, which can help us better elucidate the genetic mechanisms under-lying quantitative traits [71]. However, most dominant effects also exhibit minor effects and still require rigorous functional validation to confirm their biological functions.

### 3.2. Heritability Estimation: From Traits to SNPs

Heritability estimates are essential for evaluating the reliability and expected success of GWAS discoveries. Heritability quantifies the proportion of phenotypic variation that can be attributed to genetic factors, as it directly determines the statistical power of GWAS to detect significant loci [72]. Theoretically, for a given sample size, traits with high heritability (e.g., growth traits) exhibit stronger genetic signals and are more readily detected by GWAS, whereas traits with low heritability (e.g., reproductive traits) have genetic signals that are submerged in environmental noise, necessitating substantially larger sample sizes to achieve comparable detection power. For instance, the heritability assessments for body weight traits of Exotic goats and Boer × Central Highland goats ranged from 0.32 to 0.45 [73] and 0.00–0.50 [74], respectively. The significant heritability result emphasizes the substantial contribution of genetic factors in determining weight traits, enhancing the precision of genes identified by GWASs. However, not all traits exhibit the expected level of heritability. The heritability estimates for reproductive traits in Arsi Bale goats and Beetle goats ranged from 0.01 to 0.13 [75] and 0.03 to 0.10 [76], respectively. GWASs for traits with low heritability exhibit considerably lower statistical power given similar sample sizes. Not only do such studies struggle to detect variants with small effects, but the few significant loci they report are also more prone to overestimated effect sizes. This may partially explain why GWAS findings for reproductive traits in goats are often highly inconsistent and difficult to replicate.

To improve signal detection for these traits, research focus has shifted from estimating overall trait heritability to quantifying the contribution of individual genetic variants (such as SNP) to phenotypic variance. Estimating SNP heritability, which quantifies the proportion of phenotypic variations attributable to SNPs, has provided profound insights into the genetic architectures underlying complex traits in goats [77]. SNP heritability can provide valuable insights: for a trait with low heritability, it indicates whether its genetic architecture is primarily composed of numerous variants with small effects that remain undetected by GWAS, or whether the trait is predominantly driven by environmental factors. This informs the subsequent strategy for GWAS—whether to focus on increasing sample size or improving the accuracy of phenotypic measurement. Various method-ologies, such as LDSC [78], SumHer [79], HEELS [80], exist for estimating SNP heritability using GWAS statistical data. SNP heritability estimation models face computational challenges similar to those encountered in GWAS. Given the close relationship between SNP variation, allele frequency, and LD, adopting a more adaptable heritability model may represent an effective strategy [81].

Beyond its utility in GWAS interpretation, heritability information—particularly SNP heritability—has direct implications for genomic selection (GS), which is increasingly being implemented in goat breeding programs. Heritability estimates serve as key inputs for predicting genomic breeding values (GEBVs), influencing the accuracy of selection and the design of reference populations. For traits with low heritability, such as reproductive traits, GS offers a promising alternative to marker-assisted selection by leveraging genome-wide markers to capture small-effect loci that individually fail to reach GWAS significance. However, the practical implementation of GS in goats is hindered by limited reference population sizes and the instability of heritability estimates across breeds and environments. Addressing these gaps will require coordinated efforts to build large, multi-breed reference populations and to develop statistical models that can accommodate both additive and non-additive genetic architectures.

### 3.3. GWAS Meta-Analysis

Unlike animals such as pigs and cattle, goats are typically raised without very large-scale farming systems and exhibit vast diversity in breeds and traits. This presents a challenge, as it conflicts with the GWAS requirement for large, homogeneous datasets from uniform environments. In this context, me-ta-analysis provides a viable approach to address these limitations in goat GWAS [82]. Genetic meta-analysis can be broadly divided into two categories: multi-trait meta-analysis within a specific ancestry (such as the MTAG method), and cross-ancestry meta-analysis for a single trait [83]. By integrating GWAS summary statistics of multiple single traits, MTAG effectively corrects for spurious associations and effect size biases caused by sample overlap while simultaneously enhancing statistical power. Most importantly, for traits with high genetic correlations, MTAG can identify pleiotropic loci that jointly influence multiple traits, thereby facilitating the discovery of multiple genes that collectively affect a single trait [84,85]. Multi-ancestry meta-analysis integrates summary statistics from GWAS of the same trait across diverse populations. By pooling these data, it increases the total sample size and enhances the statistical power to detect genetic associations [86]. Although recent studies have identified numerous genetic variants and candidate genes associated with traits in goats, most of these findings are based on single breeds or populations and therefore cannot be generalized to the broader goat population. In this context, employing meta-analysis to integrate multi-breed GWAS data represents a valuable strategy for enhancing the generalizability of research findings. Furthermore, a cross-species meta-analysis involving sheep, cattle, and even humans will provide new insights into the genomic evolution of mammals.

### 3.4. Bayesian Fine-Mapping

Although GWASs have identified numerous signals associated with goat phenotypes, most of these signals do not directly contribute to the corresponding traits and are selected largely due to LD in the region. GWAS only identifies signals that are statistically associated with phenotypes. The core challenge lies in fine-mapping these signals to pinpoint the causal variants, which is essential for elucidating their biological mechanisms. Currently, the mainstream fine-mapping methods are based on a Bayesian framework [87]. Their core premise is to integrate prior probabilities with likelihood functions to calculate and compare the posterior probabilities of individual variants, thereby inferring the causal variants [88]. The calculation formula for posterior probability is:

$$P\left(Mc|D\right)=P(D|Mc)P(Mc)$$

The prior probability P(Mc) is the predefined probability of a variant being causal. Common assumptions include the independent equal probability assumption and the fixed number of causal variants assumption. The likelihood function P(D|Mc) measures how well the model Mc fits the observed data D (typically GWAS effect sizes and LD matrices). Researchers have developed several statistical fine-mapping methods, including CARMA [89], MESuSIE [90], and FINEMAP [91]. Fine-mapping has been extensively applied to identify causal variants. For example, in a GWAS of migraine using data from 967,534 individuals, fine-mapping across 102 genomic regions (encompassing 122 risk loci) identified seven variants with high posterior probability for causality [92]. In another study, a GWAS of birth weight in 3007 sheep pinpointed three causal variants within a 2.6 kb region through fine-mapping [93]. Fine-mapping, which sifts through abundant GWAS signals to pinpoint key causal variants, is an integral component of GWAS research. However, all fine-mapping methods rely on the LD between causal variants and measured SNPs, and their output depends on the preset prior probabilities. This dependence inevitably influences the results. Additionally, many candidate variants pose significant computational challenges that must be addressed.

## 4. GWAS Success in Enhancing Goat Breeding by Identifying Variation and Genes

GWAS in goats has identified a substantial number of trait-associated variants, providing key insights into underlying biological mechanisms (Table 2). Due to the complex nature of many goat traits, we adopted the classification framework outlined earlier to systematically summarize and categorize these GWAS findings.

### 4.1. Reproduction Performance

Litter size is the primary phenotype in the study of reproductive performance, characterized as a trait with low heritability that is intricately regulated by a combination of genes, each contributing subtle yet cumulative effects. GWAS is an effective method used to identify associations between genetic variation and litter size. For example, Mahmoud et al. discovered an important SNP (rs268288690) related to litter size in goats through GWAS. Notably, the mutant allelic variant (GG) of this SNP was found to exert a pronounced positive influence on enhancing litter size [49]. This SNP, situated within the confines of the GABRA5 gene, holds significant potential as a crucial molecular marker. Furthermore, we can embark on a com-prehensive analysis of the genomic regions pinpointed by GWAS, with the aim of elucidating the functional consequences of specific variations. For example, *DSCAML1* gene has been demonstrated to exhibit a robust association with reproductive characteristics in Holstein cows [125] as well as dairy goats [126]. Subsequent studies have revealed that the presence of two insertion-deletions (indels) within this gene region exerts a marked influence on litter size, establishing it as a crucial genetic marker [127].

The presence of multiple nipples is a common phenotypic trait in goats, often considered a reproductive characteristic genetically intertwined with other reproductive traits. The genetic evaluation conducted by Pauline et al. [72]. pertaining to multi-nipple traits revealed a heritability estimate ranging from 0.4 to 0.44. Following this, a GWAS was undertaken to delve into the genetic underpinnings of these traits. However, no variants reached genome-wide significance. Instead, several loci displayed suggestive associations, which may warrant further investigation in larger cohorts. This proposition indicates that the manifestation of multi-nipple traits may be intricately orchestrated by a pleiotropic interplay of genetic factors, implying the involvement of multiple genes. The prevalence of multiple genes exerting subtle influences on goat reproductive traits poses a challenge in discerning major effect genes, whereas certain signals correlated with these traits and discerned through GWAS may hint at the occurrence of pivotal regulatory events [128]. It is noteworthy that our research may have inadvertently placed undue emphasis on genetic variations residing within highly significant signals or densely linked regions in the exon region. However, regulatory events frequently occur within intronic regions, as exemplified by Liu et al.’s seminal finding that transcriptional silencing phenomena frequently manifest within the introns of actively transcribed genes [129]. On the other hand, the GWAS signal located in the edge region of LD also plays an important role in trait inheritance. For example, Gazal et al. found that SNPs in genomic regions with low LD levels often explain more heritability [130].

### 4.2. Meat Production Performance

Our argument posits that the weight and morphological traits shaping the body structure of goats serve as tangible indicators of their meat production potential. In recent years, several GWASs have been conducted to explore the genetic architecture of the weight and morphological traits in goats. Numerous genes associated with body weight traits have been identified, including *CRADD* and *HMGA2*, among others [99]. *HMGA2* orchestrates transcription processes and plays a pivotal role in modulating gene expression by inducing structural alterations within the DNA architecture [131]. Knockout studies of the *HMGA2* gene in mice [132] and pigs [133] have revealed a significant reduction in body weight of about 40% compared to their genetically intact counterparts. In addition, GWAS identified a set of novel candidate genes associated with weight traits, offering avenues for further investigation, including *PROM1* and *FBXL3*, among others [102]. Notably, *PROM1* encodes the 5-domain transmembrane glycoprotein prominin-1 (*CD133*), playing a key role in cellular self-renewal, metabolic processes, and differentiation [134]. Recent studies have shed light on the crucial role of *PROM1* in the complex structure of the retina [135]. This indicates that unraveling the causal mechanisms underlying genetic associations, when relying solely on simplistic phenotypic markers, poses a significant challenge in the realm of GWAS research. A strong genetic correlation has been observed between body shape and weight [136], exemplified by high correlation coefficients of 0.975 and 0.962 between body length and chest circumference when correlated with weight [137]. Consistent loci and candidate genes have been identified in multiple trait discoveries in GWAS research. For instance, *CNTNAP5* has emerged as a promising candidate gene, exhibiting a robust association with both body weight [138] and hip cross height [139]. GWAS investigations into eight distinct body type traits in Tashi goats have revealed significant similarities in SNPs across various traits, reinforcing the aforementioned perspective [98]. Apart from improving meat production, enhancing meat quality stands as a pivotal goal in goat breeding efforts. Selionova utilized muscle protein and fat content as phenotypic markers in a GWAS, uncovering a significant association between SNPs located at rs268269710 and the quality of goat meat [34].

### 4.3. Milk Production Performance

A QTL located on chromosome 19 (24–29 Mb) has been identified as a crucial genomic region closely associated with milk production traits. This QTL has been identified to be associated with traits such as fat content [54], protein content [108], milk production [140], breast depth [44], and breast attachment [57] in French dairy goats, British dairy goats, and New Zealand dairy goats. Interestingly, there seems to be a potential inverse relationship between production traits and breast structure within this QTL region. One possible explanation is that these distinct trait categories might be regulated by two neighboring genes or mutational variants present in this genomic segment. Conversely, the observed morphological changes in breast structure may not necessarily correlate with milk production attributes. The quality of goat milk also holds significance in GWAS research. Guan et al. successfully identified QTLs and candidate genes pertinent to protein percentage, lactose content, and dry matter content, through a combined approach of GWAS and transcriptome analysis [19]. Notably, these findings present a notable contrast with the association results reported by Martin et al. [108]. This suggests the presence of genetic heterogeneity among different goat breeds, influenced by variations in the effects of mutations occurring at different frequencies on complex traits across populations [141]. The existence of such genetic diversity among goat breeds raises concerns about the accuracy of goat reference genomes. Hence, the development of breed-specific reference genomes in goats is a worthwhile consideration [142]. In this context, undertaking a multi-breed GWAS could potentially offer a more thorough and comprehensive strategy [143].

### 4.4. Cashmere Production Performance

GWAS has been progressively advancing in pinpointing specific genetic variations and candidate genes pertaining to both cashmere yield and morphology. In a study by Rong et al., utilizing comprehensive analyses involving GWAS and haplotype construction methods, four key candidate genes crucial to cashmere yield and length have been elucidated: *HMX1*, *ADRA2C*, *AFAP1*, and *ABLIM2* [109]. Furthermore, GWAS has played a crucial role in unraveling the genetic mechanisms responsible for goat hair color. For instance, the *ASIP* gene has been identified as a pivotal factor influencing goat hair color [144], with the 13,420 bp duplication upstream of *ASIP* being deemed a necessary but not sufficient condition for this phenotype in goats [111].

### 4.5. Adaptability, Disease Resistance, and Unique Appearance Traits of Goats

Unraveling the genetic basis of adaptability in goats is pivotal for enhancing our comprehension of their ongoing fitness evolution amidst climate change. In a study by Li et al., the significant genetic locus of the *PAPSS2* gene was identified as a marker for high-altitude adaptation in goats, through a combination of GWAS and transcriptome data analysis. Subsequent gene knockout experiments underscored the critical role of the *PAPSS2* gene in cellular responses to hypoxic stress [118]. The unique horn morphology of goats is closely intertwined with their adaptability to diverse environmental conditions. In addition, the breeding of hornless goats has gained popularity among breeders due to practical reasons such as reduced injury risks and ease of handling during production. GWAS plays a vital role in breeding goat populations with hornless traits. For instance, Zhang et al. utilized GWAS to pinpoint three SNPs associated with the hornless phenotype on Chromosome 1 (Chr1:129789816, Chr1:129791507, and Chr1:129791577) [120]. In contrast to horns, the meat drape characteristic of goats offers insights into their evolutionary trajectory. Reber et al. uncovered a strong correlation signal through GWAS, linked to limb development and growth processes in goats [123]. While GWAS has made significant strides in disease research, its primary focus remains on pivotal diseases affecting goats, notably brucellosis [115].

## 5. Problems and Countermeasures of Goat Genetic Structure Research Based on GWAS

**Figure 3:**
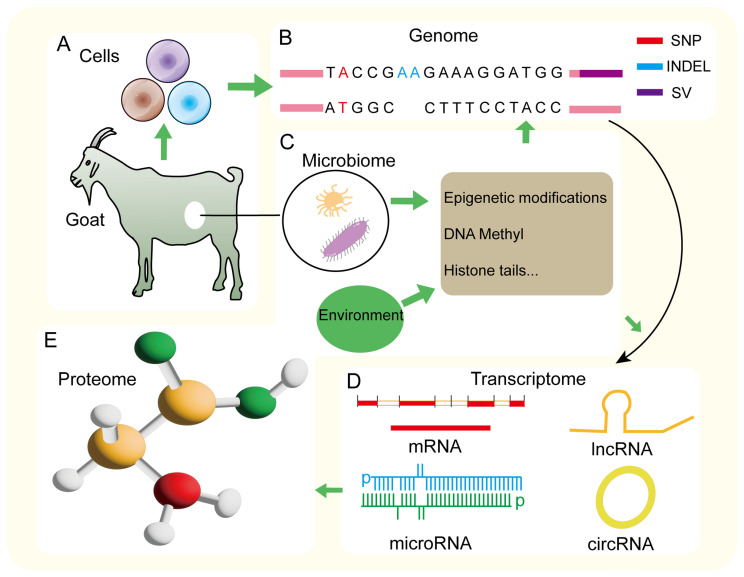
The biological mechanisms at the multi-omics level that influence trait expression in goats. (**A**) Goat phenotypes are classified into terminal phenotypes and intermediate phenotypes. (**B**) Genomic variation is the basis for trait expression. (**C**) Epigenetic modifications caused by microbial and environmental regulation affect gene expression. (**D**) At the transcriptome level, non-coding RNAs (such as miRNA, circRNA, and lncRNA) affect protein translation by regulating mRNA expression. (**E**) Proteomic level.

In the two decades following the groundbreaking publication of the first GWAS research article, a vast repository of thousands of relevant gene loci has been meticulously mapped across various species, encompassing humans, animals, and plants. However, the intricate biological mechanisms underlying these associated signals and phenotypic traits remain largely unexplored and inadequately elucidated. In goats, phenotypes can be conceptually categorized based on their proximity to the ultimate biological functions into terminal phenotypes (such as milk yield, growth rate, disease resistance, and other individual or population-level traits) and intermediate phenotypes (such as gene expression patterns, protein abundance, metabolite profiles, and other molecular and cellular-level traits) (Figure 3A). The genetic basis of these phenotypes ultimately originates from genomic variations (e.g., SNPs, Indels, SVs, etc.) (Figure 3B). These variations collectively form a finely regulated biological network by influencing RNA transcription, splicing, stability, and ultimately protein synthesis, modification, and function, thereby determining phenotypic manifestation (Figure 3D,E). Additionally, epigenetically regulated modifications (e.g., DNA methylation and histone modifications) induced by environmental factors, microbial interactions, and other influences directly affect the activity of the genome and transcriptome, profoundly participating in and regulating the molecular networks (Figure 3C). In addressing the elucidation of molecular mechanisms underlying complex traits in goats, integrating multiple types of omics data serves as a comprehensive and effective approach, and represents a key priority for future research.

### 5.1. The Integration of Diverse Genetic Variation Types Contributes to Elucidating the Heritability Missing Observed in GWAS

The convenience of detection methodologies coupled with the abundance of loci serves as the cornerstone for the prevalence of SNPs as a focal point of research. For instance, the occurrence frequency of SNPs in mammals is approximately 0.1%, highlighting their prevalence and importance in genetic studies [145]. Nonetheless, SNPs often account for merely a fraction (ranging from 2% to 15%) of genetic variability underlying many complex traits. As whole-genome sequencing technology advances, the identification of a broader spectrum of genetic variation types promises to enhance the understanding of phenotypic diversity. By virtue of their composition as amalgamations of multiple tightly linked alleles, haplotypes possess the capacity to discern associations that remain elusive to individual SNP. In their diverse GWAS in barley, Lorenz et al. found that haplotype-based GWAS showed increased efficacy in detecting QTL [146]. Copy number variation (CNV), which is closely linked to gene expression patterns and phenotypic traits, plays a crucial role in phenotypic diversity [147]. In a recent study, Huang et al. conducted a CNV-based GWAS across three ruminant species—cattle, goats, and sheep—revealing Copy Number Variation Regions (CNVRs) shared across these species exhibited greater consistency compared to SNPs [148]. While the overall impact of structural variation (SV) and short tandem repeat sequences (STRs) on complex traits remains incompletely understood, there is evidence suggesting correlations between these genetic features and the manifestation of such traits. Genomic feature analysis by Jakubosky et al. on SV and STR demonstrated a significant of these genetic elements in their association with traits identified through GWAS [149].

### 5.2. Multi Omics Joint Analysis Helps to Understand Genetic Structure

GWAS have successfully linked thousands of genomic loci to complex traits, yet a persistent challenge remains in elucidating the underlying causal relationship between these genes or associated loci and their corresponding phenotypic manifestations. This challenge has stimulated the development of various omics methodologies aimed at elucidating the intricacies of genetic structure. Building upon the foundation set by GWAS, a series of innovative methodologies have emerged, including Transcriptome-Wide Association Studies (TWAS) [150], epigenetics-wide association study (EWAS) [151], and proteome-wide association study (PWAS) [152], all operating within this methodological framework. TWAS integrates genetic regulation expression (GReX) with GWAS to develop a GreX model, offering insights into the potential correlation between gene expression and traits. This represents a pivotal approach for the integration of functional genomics with GWAS [153]. Mapel et al. identified a significant correlation between splicing events of SPATA16 and fertility through TWAS and co-localization analyses in bull testicular tissue [154]. Epigenetic modifications, prevalent at the transcriptome level, play pivotal roles in shaping the genetic architecture of genes. EWAS represents a methodology designed to capture epigenetic variations that are intricately associated with complex traits, offering a complementary perspective to GWAS. For instance, an EWAS by Lu et al. in mammalian systems revealed a notable correlation between the mutation status of HTT and the onset of Huntington’s disease [155]. PWAS, akin to TWAS, establishes a comprehensive genetic prediction model customized for individual proteins. In a pioneering study, Zhu et al. identified 16 novel protein biomarker candidates closely associated with pancreatic ductal adenocarcinoma (PDAC) using PWAS [156]. Expanding GWAS into other omics fields holds the potential to enhance our understanding of genetic architecture influencing phenotypic variation. Furthermore, Integrating and analyzing ex-tensive datasets across various omics domains is poised to yield more pro-found insights. For instance, Schlosser et al. combined TWAS and PWAS methodologies to compile a comprehensive list of putative causal target genes, tissues, and proteins relevant to kidney function and damage [152].

### 5.3. Environmental Factors Affect Complex Traits

It is widely recognized that complex traits are influenced by both genetic and environmental factors, as well as their intricate interactions (G × E) [157]. Leveraging the advancements in Mendelian randomization (MR), the identification of G × E interactions can be facilitated through the implementation of level pleiotropy tests within the MR framework. This methodology has been successfully employed in a bibliometric study conducted by the Global Lipid Genetics Alliance, aiming to reveal genetic loci underlying the complex interplay between smoking, alcohol consumption, and lipid traits [158]. The feeding environment significantly impacts the dietary preferences of goats, prompting an exploration into the intricate (G × E) effects related to dietary selection to enhance production efficiency. Walker et al. investigated the varying tendencies of goats in selecting juniper as a food source, analyzing the influence of both genetic predispositions and environmental stimuli on this specific selection behavior [159]. In addition, certain genetic signals unveiled through GWAS have the potential to impact the microbial landscape of the human body, subsequently influencing the manifestations of intricate phenotypic traits [160]. Microbiome-Wide Association Studies (MWAS) represent a powerful approach to uncover microbial signatures intricately linked to the expression of complex traits [161].

## 6. Perspectives of GWAS in Goat

Over the past decade, extensive GWASs on goat genomes revealed a rich array of genetic variants closely linked to diverse phenotypic traits. Firstly, the remarkable advancements in phenotype measurement technology have significantly mitigated challenges associated with quantifying intricate phenotypic traits in goats. Secondly, the evolution of sequencing methodologies has enhanced our ability to detect genetic variations within the goat genome. Lastly, improved statistical models and computational capabilities have expedited GWAS execution. Together, these advancements strongly support the use of GWAS in goat breeding. In the realm of goat genomics, fine-mapping techniques enable precise localization of QTL and SNP associated with traits identified in GWAS [162]. Meanwhile, the integration of multi-omic analyses and the investigation of gene-environment interactions (G × E) offer a promising avenue to uncover the heritability attributed to previously undetected genes. This effort may lead to a transition from GWAS to Omic-Wide Association Studies (OWAS). The growing capabilities of deep learning models in artificial intelligence (AI) are poised to expand and enhance the application and depth of GWAS. Therefore, prioritizing the development of AI-powered deep learning models tailored for enriching the scope and precision of GWAS in goats is crucial.

## Figures and Tables

**Table 1:** Progress in GWAS-related statistical models.

Model	Application of Goat				Time	Author
	Breed	Number	Trait	Ref.		
MLM	Dairy goat	208	Milk production	[36]	2006	[33]
EMMA	Markhoz goat	228	Cashmere	[37]	2008	[38]
EMMAX	—	—	—	—	2010	[39]
Compressed LMM	—	—	—	—	2010	[40]
Fast LMM	—	—	—	—	2011	[41]
GEEMA	Murciano-Granadina goat	825	Body conformation	[42]	2012	[43]
MLMM	Dairy goat	2381	Milk yield and conformation	[44]	2012	[45]
MTMM	—	—	—	—	2012	[46]
Farm CPU	Chubao black-head goat	500	Growth and reproduction	[47]	2016	[48]
BLINK	Markhoz goat	136	litter size at birth and weaning	[49]	2019	[50]
Fast GWA	—	—	—	—	2019	[51]

—: Missing data.

**Table 2:** List of diverse traits dissected via GWAS in goat.

Trait	Breed	Sample Number	Significant Marker Count	Ref.
Litter size	Markhoz goat	136	4	[49]
Litter size	Dazu black goat	150	18	[94]
Litter size	Youzhou black goat	206	1	[16]
Litter size	Jabal Akhdar Omani goat	72	8	[95]
Litter size	Three breeds	336	17	[96]
Litter size	Arbas cashmere goat	361	6	[97]
Eight Body conformation	Tashi goat	155	385	[98]
Weight	Karachai goat	287	11	[34]
Seven body conformation			7	
Body weight	Karachai goat	269	5	[99]
Seven body conformation			60	
Body conformation	Zhongwei goat	240	342	[100]
Carcass	South African goat	73	40	[101]
Body weight	Inner Mongolia cashmere goat	1920	21	[102]
Milk production	Murciano-Granadina goats	660	19	[103]
Milk quality	Karachai goat	167	43	[104]
Udder conformation	Dazu black goat	150	10	[94]
Milk production	Alpine, Saanen goat	1707	146	[105]
Udder conformation			10	
Udder conformation	New Zealand goat	1058	27	[57]
Milk yield trait	French dairy goat	1114	457	[106]
Milk production	American Alpine Goat	72	30,594	[107]
Milk yield and somatic cell score	New Zealand dairy goat	3732	43	[54]
Seven milk production	Murciano-Granadina goat	822	24(QTL)	[19]
Milk yield	Saanen, Toggenburg, Alpine	2381	2	[44]
Udder conformation		402	3	
Milk production trait	French dairy goat	2209	2(QTL)	[108]
Supernumerary teat	Alpine, Saanen goat	2254	17	[72]
Cashmere yield	Inner Mongolia cashmere goat	404	28	[109]
Cashmere morphology			123	
Cashmere morphology	Northwest Xizang White Cashmere Goat	539	151	[110]
Cashmere yield			60	
Coat color	Jintang black goat	65	660	[111]
Cashmere morphology	Inner Mongolia Cashmere goat	192	78	[112]
Cashmere yield			52	
Coat color	Markhoz goat	228	116	[37]
Cashmere morphology			31	
Cashmere diameter	Cashmere goat	436	26 (QTL)	[113]
Coat color	Valais Blacknecked and Coppernecked goat	45	3	[114]
Brucellosis infection	Damascus goat	96	10	[115]
*Haemonchus contortus* infection	Multiple breed	144	2	[116]
Gastrointestinal nematode infection	Creole goat	182	7	[117]
Adaption	Tibetan and other goat	156	250	[118]
Resilience	UK dairy goat	10,620	7	[119]
Polledness	Saanen dairy goat	106	3	[120]
Polledness	Jintang black goat	45	14	[121]
Polledness	Australian goat	175	10	[122]
Wattle	Swiss goat	341	2	[123]
Juniper consumption	Boer × Spanish and Angora	711	571	[124]

## Data Availability

The original contributions presented in this study are included in the article. Further inquiries can be directed to the corresponding author.
